# Solving Static and Dynamic Disorder in Cu_4_TiTe_4_: Crystal Structure and Thermodynamic Properties

**DOI:** 10.1021/acs.inorgchem.4c04585

**Published:** 2025-02-07

**Authors:** Jorge Suárez-Recio, Álvaro Lobato, Fernando Izquierdo-Ruiz, Ruth Franco, Alberto Otero-de-la-Roza, J. Manuel Recio

**Affiliations:** † Instituto de Fusión Nuclear “Guillermo Velarde”, Universidad Politécnica de Madrid and Departamento de Ingeniería Energética, Universidad Politécnica de Madrid, Madrid E-28006, Spain; ‡ MALTA-Consolider Team and Departamento de Química Física, Universidad Complutense de Madrid, Madrid 28040, Spain; § MALTA-Consolider Team and Departamento de Química Física y Analítica, 16763 Universidad de Oviedo, Oviedo 33006, Spain

## Abstract

Cu_4_TiTe_4_ shows positional disorder because one of the copper atoms does not occupy a precise position in the unit cell. This fact complicates the development of simple and reliable crystalline models capable of capturing the promising thermodynamic and optical properties of Cu_4_TiTe_4_. Here, we select practical supercells accounting for the different Cu atomic environments in the crystal and identify nonequivalent structural configurations. Their electronic energies and thermodynamic properties are calculated by coupling DFT and the quasi-harmonic approximations. Average values corresponding to the experimentally observed Cu_4_TiTe_4_ structure are obtained introducing Boltzmann weights based on the total energy of these configurations. For Cu_4_TiTe_4_, differences in the calculated properties among the 16 nonequivalent configurations of its 2 × 2 × 1 supercell demonstrate the inadequacy of focusing just on a single configuration. After calculating the energy barriers associated with the diffusion of the disordered copper atoms among its four equivalent positions (lower than 0.5 eV), we evidence the importance of taking into account this dynamic disorder, which reveals a negative thermal expansion for this telluride at low temperatures, not found under the static disorder approach.

## Graphical abstract

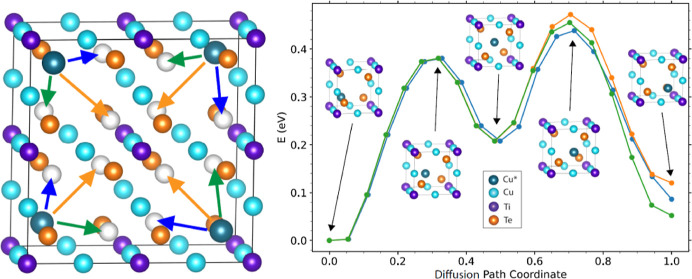

## Introduction

Behind the apparent conventional composition, Cu_4_TiTe_4_ hides a rich structural landscape due to the positional disorder of one of its copper atoms. In fact, the compound would be better named Cu_3_Cu^★^TiTe_4_ to highlight the copper atom that can occupy any one of the four Wyckoff equivalent positions assigned to it in the experimental thermally averaged cubic *P*4̅3*m* space group.^1^ This positional disorder introduces attractive chemistry and a number of technological functionalities to this and other copper metal chalcogenides, being their narrow bandgap and low thermal conductivity specific properties of interest for solar cells and thermoelectric applications, respectively.^2–5^ From the theoretical point of view, the proposal of rigorous computational models able to deal with these fractional atomic occupations in crystals constitutes a very demanding challenge if the objective is to advance in the understanding of their properties. See for example an illustrative and short review of different alternatives by Grau-Crespo et al. and references therein.^6^

Positional disorder is a particular case of the more common substitutional disorder found in solid solutions. For example, in the title compound, the disorder could be seen as due to a mixture of one atom of Cu^★^ and 3 atoms of *nothing* (vacancies) sharing a Wyckoff position with multiplicity four. In general, the different atomic species are more or less randomly distributed over the shared equivalent positions, which breaks the translational periodicity of the crystal. Since the time scale of the atomic movements (∼10^–12^ s) is faster than that of the standard X-ray diffraction experiments (∼10^–4^ to 10^–5^ s),^7^ the resulting X-ray diffraction patterns provide information on the average structure of the disordered crystal. From a computational point of view, modeling this average structure can constitute an enormous task since, in principle, it requires considering all possible structural configurations.

It is common to distinguish between two types of positional disorder. The first one accounts for the so-called static disorder in which the positions of the disordered atoms are fixed at a distribution given by the thermal equilibrium configuration population found at the synthesis temperature *T*_s_. For Cu_4_TiTe_4_, the synthesis procedure involves several annealing and quenching steps after heating the samples at temperatures as high as *T*_s_ = 900 K.^8^ If only static disorder exists, the equilibrium distribution of the structural configurations at 900 K must be considered to model the properties of Cu_4_TiTe_4_ at all temperatures. Second, dynamical disorder occurs when the energy barriers of the disordered atoms diffusing among the equivalent Wyckoff position are low. This means that thermodynamic equilibrium is achieved with a distribution of Cu and vacancies that is temperature dependent. Accordingly, the dynamic disorder has to be realistically modeled by including the temperature dependence of the Boltzmann distribution in the evaluation of the thermodynamic properties.

Even if only the static type of disorder (temperature independent) is considered, a realistic simulation of the positional disorder can be computationally very expensive, should we follow a first-principles quantum mechanical strategy to evaluate the energy of the nonequivalent configurations due to the loss of translational symmetry caused by the atomic disorder. Several possibilities have been proposed. In inorganic crystals as Cu_4_TiTe_4_, the virtual crystal approximation (VCA) implemented in CASTEP^9^ constitutes a practical procedure successfully applied for example in PbTi_0.5_Zr_0.5_,^10^ (BiScO_3_)_1–*x*_–(PbTiO_3_)_*x*_,^11^ hollandite-type KAlSi_3_O_8_ and gehlenite Ca_2_Al_2_SiO_7_ with Al/Si disordered atoms,^12^ but shows also theoretical and technical limitations preventing its use in combination with partial atomic occupations.^9^ In contrast with the VCA formalism, a direct selection of supercells accounting for the positional disorder seems to be the best modeling framework provided the number and size of the supercells do not result in an unfeasible amount of calculation. This is in agreement with Dittrich, who noticed that since real atoms and molecules cannot split in fractions, a number of different *archetypes* structures should be involved in the modelization of a disordered structure.^13^

Computational strategies based on supercell calculations introduce limitations due to the large number of configurations involved if we seek a realistic model of the disordered crystal. Todorov et al.^14^ partially reduced the configurational space by comparing energies and space groups. Grau-Crespo et al.^6^ emphasized the computational cost of such a rigorous procedure even if a quantum mechanical first-principles methodology is not followed. In their paper, they carefully addressed the selection of the reduced set of nonequivalent structural configurations within a given supercell “taking advantage of the crystal symmetry of the lattice”. Using their strategy, the authors successfully modeled the thermodynamics of the paramagnetic FeSbO_4_ static disordered crystal. Nevertheless, Grau-Crespo et al. did not discuss the possibility of atomic mobility, and, therefore, the thermodynamic properties considering dynamic disorder were not evaluated. Another alternative that reduces the configurational space just to a single configuration was pioneered by Zunger et al. in the nineties through the so-called special quasirandom structure (SQS) model.^15,16^ For a given supercell size, the SQS is generated requiring that its radial distribution function matches as much as possible that of the true random disordered solid. Although this formalism has been improved and successfully applied to alloys and doped systems,^17,18^ its implementation to model temperature-dependence of thermodynamic properties has not been explored yet.

In this article, we follow a general three-step computational strategy to model positional disorder that starts by defining the size of a practical supercell and the identification of its reduced set of nonequivalent structural configurations. In the Cu_4_TiTe_4_ compound explored here, 16 and 217 configurations constitute this reduced space when 2 × 2 × 1 and 2 × 2 × 2 supercells with 256 and 65,536 Cu–Ti–Te atomic arrangements are considered, respectively. In the second step, the electronic energies and the thermodynamic properties of those configurations within the reduced set are obtained by combining DFT calculations and the quasi-harmonic approximation. Lattice parameters, equations of state, and several thermal properties, such as volumetric thermal expansion, heat capacities, or entropy have been evaluated in the range of 0–400 K for Cu_4_TiTe_4_. Differences among the 16 nonequivalent configurations of the 2 × 2 × 1 supercell reveal the inappropriateness of studying just a particular configuration. In this regard, it could be interesting to examine in an independent work how the SQS model performs in this situation, since our system can be also described as a solid solution with stoichiometry Cu_12_Cu_4_^★^□_12_Ti_4_Te_16_ compatible with a 2 × 2 × 1 supercell. Finally, averaged thermodynamic properties can be calculated using either a fixed *T*_s_ temperature (static disorder) or temperature-dependent Boltzmann weights (dynamic disorder). Expressions for both schemes are derived paying special attention to those properties involving derivatives with respect to temperature. After computing the energy barriers associated with the diffusion of Cu^★^ among the four equivalent positions (lower than 0.5 eV), we saw it necessary to take into account the dynamic disorder, which unveils a negative thermal expansion for this telluride at low temperature not found under the static disorder approach.

The rest of this article is divided into three more sections. The next section presents our thermodynamic model to deal with static and dynamic positional disorder in crystals, its implementation in the case of the Cu_4_TiTe_4_ crystal, and the computational parameters used in the electronic structure and phonon dispersion calculations. We then analyze in two separated subsections the results obtained, respectively, in the nonequivalent structural configurations of the 2 × 2 × 1 supercell of Cu_4_TiTe_4_ and those calculated after applying the equations derived from our static and dynamic thermodynamic model. The paper ends by summarizing the outcome of the discussion and the main findings.

## Thermodynamic Modeling of Positional Disorder

### Basic Equations for Static and Dynamic Disorder

We aim to model the thermodynamic properties of a crystalline solid exhibiting positional disorder due to an atomic species A^★^ that occupies fewer positions than its Wyckoff’s position multiplicity (*N*_W_). The thermodynamic properties will be calculated following a first-principles DFT methodology for the electronic energy and accounting for the thermal contributions through the quasi-harmonic approximation (QHA) (see below). The first important decision concerns the definition of the unit cell where we will apply the DFT + QHA strategy. We present different crystalline models with computational complexity increasing with the number of atoms in the corresponding unit cell. Let us consider the common case in which the unit cell has one formula unit (*Z* = 1) and only one of the *N*_*W*_ positions is occupied by A^★^. To build the simplest crystalline model, we choose the unit cell usually determined in X-ray experiments. Obviously, since all the *N*_W_ positions are equivalent, DFT + QHA provides the same results regardless of the position in which *A* ^★^ is located. However, this is not a good model describing the positional disorder because by replicating this unit cell only one of the equivalent Wyckoff positions is always occupied hindering the observed positional disorder from being reproduced in the crystal. The resulting structure therefore is inconsistent with experiment, for instance, it has lower symmetry.

A more realistic alternative is to build a supercell model with *Z* = *N*_W_. The size of this supercell allows it to populate all the Wyckoff positions associated with A^★^ in a manner that is consistent with the experimentally observed occupations. This supercell is made of *N*_W_ subcells with *Z* = 1. Notice that the number of different ways to carry out this assignment is $N_{W}^{N_{W}}$ , provided one and only one A^★^ atom is located in each of the subcells. In the general case of having a supercell with *Z*_s_ formula units, the configurational space would contain $N_{W}^{Z_{s}}$ structures. Although the size of the supercell can be increased, *Z* = *N*_W_ is a reasonable choice, being the smallest supercell allowing a realistic modeling of the positional disorder. Many of the $N_{W}^{Z_{s}}$ possibilities are symmetrically equivalent and yield the same DFT + QHA results. Classifying the nonequivalent configurations (*n*_c_) in terms of the number of equivalent configurations or degeneracies (*g*_i_) associated with each of them is important to reduce computational time. We used the structure comparison tool in the program^19^ to perform this operation, which works in a similar way as other codes such as SOD.^6^ Applying this procedure, we arrive at a model for positional disorder comprising *n*_c_ configurations, each of them with a degeneracy *g*_i_ and with the number of formula units in their unit cell equal to the multiplicity of the Wyckoff position in which A^★^ shows positional disorder.

Let us now consider the *n*_c_ configurations (*C*) with electronic energy *E*_C_ at each of their own equilibrium volumes *V*_C_ under a pressure *p*. In the isothermal–isobaric ensemble, each configuration corresponds to a region in phase space clustered around their equilibrium geometry at the considered pressure. The isothermal–isobaric partition function can therefore be grouped by configurations

1 $$\Delta \left(p,T\right)=\sum_{k}e^{-\left(E_{k}\left(V\right)+pV\right)/\left(k_{B}T\right)}=\sum_{C}^{n_{c}}g_{C}\sum_{k}^{C}e^{-\left(E_{k}\left(V\right)+pV\right)/\left(k_{B}T\right)}$$

where *E*_k_ is the total energy (electronic plus vibrational) of the system in vibrational state *k*, *g*_C_ is the multiplicity of configuration *C*, and the inner sum in the right-hand side sums only over the states belonging to that configuration. Since the partition function is related to the Gibbs energy of the system, we can also write

2 $$e^{-G/k_{B}T}=\sum_{C}g_{C}e^{-G_{C}/\left(k_{B}T\right)}$$

where *G* is the (average) Gibbs energy of the disordered system and *G*_C_ is the free energy corresponding to configuration *C*. The latter can be calculated using the standard techniques like DFT + QHA. For a system with dynamic disorder in thermodynamic equilibrium, the probability of observing configuration *C* is given by

3 $$w_{C}=\frac{e^{-G_{C}/\left(k_{B}T\right)}}{\sum_{D}^{n_{C}}g_{D}e^{-G_{D}/\left(k_{B}T\right)}}=e^{-\left(G_{C}-G\right)/\left(k_{B}T\right)}$$

where the Gibbs energy of each configuration is given by

4 $$G_{C}=E_{C}+pV_{C}+F_{\text{vib}}^{C}$$

with *E*_C_ and *V*_C_ the equilibrium energy and volume at pressure *p* and *F*_vib_^C^ the vibrational contribution to the free energy (for simplicity, we disregard other contributions). In this work, we make the reasonable assumption that the vibrational contribution is similar for all disordered configurations. Under this approximation and if we are at *p* = 0, the configuration probabilities in eq 3 reduce to the familiar Boltzmann weight

5 $$w_{C}=\frac{g_{C}e^{-E_{C}/\left(k_{B}T\right)}}{\sum_{D}^{n_{C}}g_{D}e^{-E_{D}/\left(k_{B}T\right)}}$$

In addition, the vibrational contribution weights to the free energy and the entropies of all configurations are equal. The configuration weights can be used to calculate the average of any mechanical property in the system (i.e., those that can be defined for each state) in the usual way. For instance

6 $$U=\sum_{C}w_{C}E_{C}$$

7 $$V=\sum_{C}w_{C}V_{C}$$

On the other hand, nonmechanical properties have an additional contribution from configurational dynamic-induced disorder, which arises from the temperature dependence of the configuration weights. Because *G* is the thermodynamic potential in the isothermal–isobaric ensemble, the correct procedure for calculating averages in the case of a nonmechanical property can be obtained by differentiation of eq 2. As an example, in this work, we examine the importance of taking dynamic disorder into account for the thermal expansion coefficient (α). It is easy to show by differentiation of eq 7 under the stated assumptions that

8 $$\alpha =\frac{1}{V}{\left(\frac{\partial V}{\partial T}\right)}_{p}=\sum_{C}w_{C}\frac{V_{C}}{V}\left[\alpha_{C}-\frac{U-E_{C}}{k_{B}T^{2}}\right]$$

with $\alpha_{C}=\frac{1}{V_{C}}{\left(\frac{\partial V_{C}}{\partial T}\right)}_{p}$ .

In the case of a crystal with static disorder, the weight of each configuration is given by the synthesis temperature of the material, and, therefore, the *w*_C_’s in the above equations are constant and independent of the temperature at which the thermodynamic property is evaluated. In other words, in this case, we assume that the material is quenched keeping the same configuration populations as the ones obtained at the synthesis temperature (*T*_s_), which leads to

9 $$X=\sum_{C}^{n_{C}}w_{C}\left(T_{s}\right)X_{C}$$

for every property, with *T*_s_ the (fixed) synthesis temperature.

On the contrary, the dynamic disorder appears when the energy barrier for the diffusion of A^★^ between its equivalent Wyckoff positions is low enough to allow atomic hoping. The thermodynamic equilibrium is observed if diffusion times connecting all the configurations are shorter than experimental measurement times. In this case, the *w*_C_ factors must be evaluated at the same temperature as the thermodynamic property to account for this dynamic disorder.

In summary, the flowchart of the computational strategy consists of three steps. It starts by defining the size and parameters of a suitable supercell able to account for the different positions that the A^★^ atom can occupy. Once the nonequivalent configurations have been identified, the second step involves DFT + QHA calculations in all of them. Finally, averaged thermodynamic properties of the positional disordered crystal (whether static or dynamic) are evaluated thanks to the operative expressions given in the above equations. In Figure 1, a schematic description of the procedure is presented for the static case.

**Figure 1:**
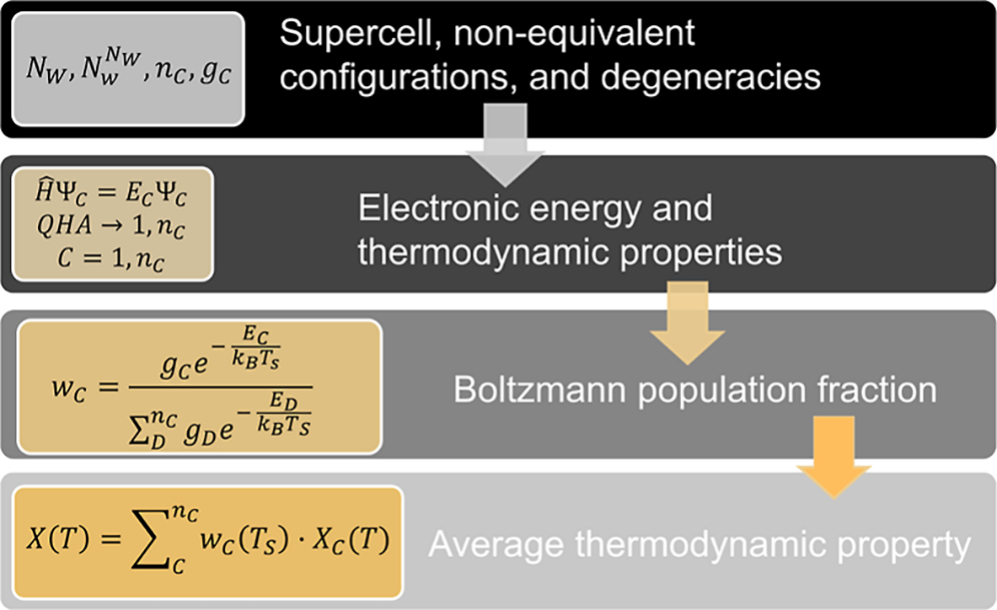
Flowchart summarizing the steps of the computational strategy proposed to evaluate thermodynamic properties in the case of static positional disorder. Symbols and equations are described in the main text.

### Crystalline and Disorder Modeling in the Case of Cu_4_TiTe_4_

The structure of Cu_4_TiTe_4_ is best described by analogy with the mineral sulvanite (Cu_3_VS_4_).^4^ In this mineral, atoms in well-defined positions conforming to an ordered crystalline structure occupy the cubic unit cell. V atoms are located at the corners of the cell, Cu at the middle points of all the edges, and S atoms in half of the tetrahedral interstices. In Cu_3_Cu^★^TiTe_4_, Ti plays the role of V, Te plays the role of S, and only the extra Cu^★^ atom shows a new position in the unit cell introducing positional disorder since it occupies one out of the other four equivalent tetrahedral interstices of the cubic cell. These positions are labeled with the letters A, B, C, and D [see Figure 2 (left)].

**Figure 2:**
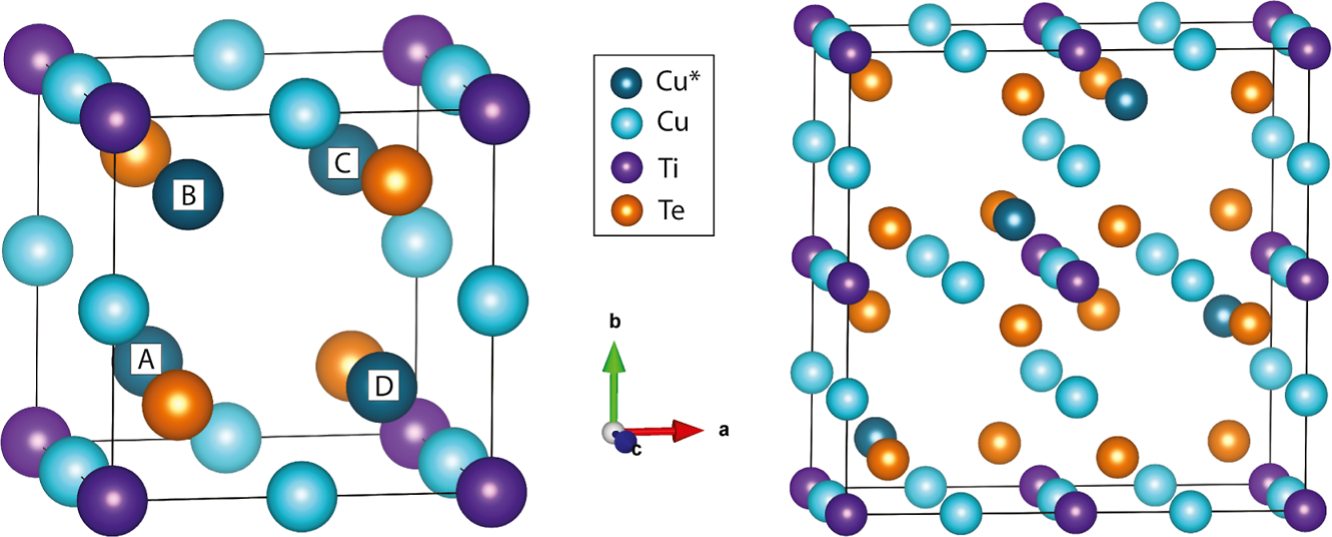
(Left) The 1 × 1 × 1 cubic unit cell of Cu_4_TiTe_4_ highlighting the four positions of the special Cu^★^ atom with the labels A, B, C, and D. (Right) One of the 16 nonequivalent 2 × 2 × 1 supercells (ADBC) of Cu_4_TiTe_4_.

In the experimental dynamically averaged cubic cell with *P*4̅3*m* symmetry, there is only one formula unit and Cu^★^ can be found in any of the four tetrahedral interstices with the same probability, i.e. fractional occupation equal to $\frac{1}{4}$ . Using the notation introduced in the previous subsection, *N*_W_ = 4 since Cu^★^ occupies the 4e Wyckoff position in the unit cell. A 2 × 2 × 1 supercell with four formula units (*Z* = *N*_W_) constitutes the smallest possible crystalline model capable of describing the positional disorder of Cu^★^ atoms. This supercell contains 16 tetrahedral interstices where the four Cu^★^ atoms are distributed with the restriction of no more than one Cu^★^ atom in each of the four subcells in which the supercell is divided [see Figure 2 (right)]. Depending on which of the A, B, C, and D positions are occupied by the Cu^★^ atom in the four subcells, we arrive at the 4^4^ = 256 different configurations of this 2 × 2 × 1 supercell. In Figure 3, we schematically identify the set of the 16 configurations that are chemically nonequivalent.

**Figure 3:**
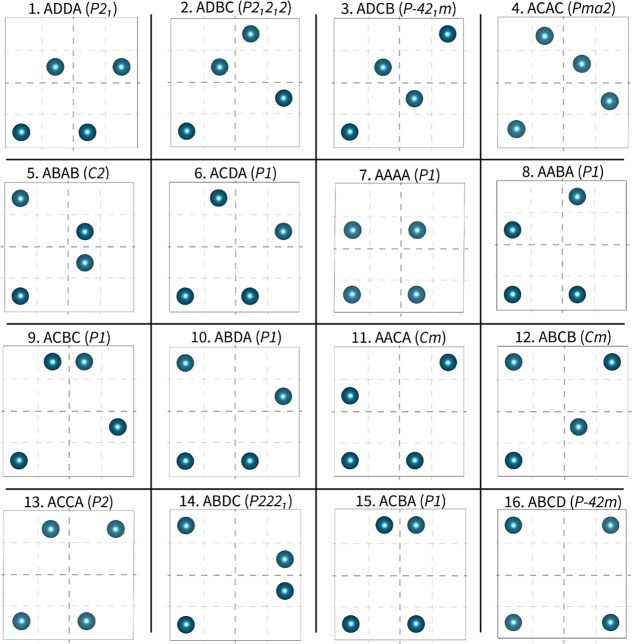
Schematic view of the positions of the four Cu^★^ atoms in the 16 nonequivalent 2 × 2 × 1 supercells of Cu_4_TiTe_4_. A four-letter code is assigned to each supercell according to the positions of their four Cu^★^ atoms. The assigned numbers (1–16) rank the structures in order of increasing energy. Space groups in parentheses.

Notice that the next supercell with a size compatible with the composition and positional disorder of the title compound is a 2 × 2 × 2 cell and involves 65,536 configurations, being 217 chemically nonequivalent. This number and the number of atoms in the cell (72) makes the 2 × 2 × 2 supercell computationally prohibitive, though calculations were also performed in selected configurations of this supercell to check consistency with the results in the 2 × 2 × 1 supercell that will be selected from now on to model the positional disorder in Cu_3_Cu^★^TiTe_4_.

### Computational Parameters

All electronic structure calculations in this paper were performed in the density-functional theory (DFT) framework^20^ using the plane-wave Vienna ab initio simulation package (VASP).^21^ Projector augmented wave (PAW) data sets^22^ were used under the generalized gradient approximation (GGA) and the Perdew–Burke–Ernzerhof (PBE) scheme.^23^ The Cu data set contains 11 valence electrons in the valence band, corresponding to the ground state configuration 3d^10^ 4s^1^. The Ti and Te data sets were also characterized by their conventional 3d^3^ 4s^1^ and 5s^2^ 5p^4^ valence electronic configurations, respectively. The plane wave cutoff energy was set at 350 eV, and optimized geometries for all configurations were obtained using the conjugated gradient algorithm implemented in VASP. The convergence threshold for the forces in the geometry optimization was considered to be achieved when the Hellmann–Feynman forces on the atoms did not exceed 0.03 eV Å^–1^.

Supercell calculations were performed to simulate Cu^★^ positional disorder. 2 × 2 × 1 and 2 × 2 × 2 Cu_4_TiTe_4_ supercells were generated automatically with an in-house script, resulting in the previously mentioned 256 and 65,536 configurations, respectively. The duplicates in the list of structures were pruned using the crystal comparison tool implemented in critic2,^24^ based on the powder-diffraction-based similarity index proposed by de Gelder et al.^25^ For each of the 16 nonequivalent configurations of the 2 × 2 × 1 supercell, a data set of 15 *E*–*V* points was calculated in a volume range from 160 to 240 Å^3^. The zero pressure equilibrium volume (*V*_0_), bulk modulus (*B*_0_), and its zero pressure derivative (*B*_0_^′^) were obtained by a Vinet equation of state fitting using the Gibbs2 code.^26,27^

Phonon frequency calculations were carried out using the finite displacement method implemented in the phonopy program,^28^ at all points in the volume grid for the minimum-energy configuration. A 2 × 2 × 2 *q*-point mesh was used for calculating the vibrational frequencies, and the phonon density of states was obtained using a Fourier reinterpolation on a 20 × 20 × 20 grid. The resulting phonon density of states curves, together with the energy-volume data, were used in Gibbs2 ^26,27^ to calculate the thermodynamic properties as a function of temperature at zero pressure.

The analysis of the atomic diffusion is performed using the nudged elastic band (NEB) method, as implemented in VASP.^29^ The NEB method finds the minimum-energy transition path between two equilibrium configurations. The method is used to identify the most favorable trajectory that the Cu^★^ atom follows when it moves between two equivalent positions within the same quadrant of the supercell. The energy barrier is calculated by subtracting the total energy at the saddle point from that at the initial equilibrium configuration. For each migration, a total of 9 images (1–9) are initially generated, with the first and last corresponding to the equilibrium configurations. The initial coordinates of all atoms in the intermediate images 2–8 are set to values linearly interpolated between images 1 and 9. In our NEB calculations, each image M is linked to the (M-1) and (M+1) images with an interimage string constant of 5.0 eV/Å^2^ and a maximum allowed translation step of 0.25 Å. The multi-image minimization procedure is processed using the force-based fast inertial relaxation engine (FIRE) method^30^ until a predetermined tolerance value for the force of 0.1 eV/Å is reached.

## Results and Discussion

### Sixteen Independent Configurations

One of the central questions in this study is how important the variations of structural, energetic, electronic, and thermodynamic properties are among the position-disordered configurations. We selected various random nonequivalent configurations of the reduced set of the large 2 × 2 × 2 supercell (L) and performed a systematic analysis of the 16 nonequivalent configurations of the 2 × 2 × 1 small supercell (S). Relative to the S supercell, computational times are roughly quadruplicated in the L supercell, which makes this option prohibitive if we aim to perform the same systematic study within its configurational space of 256 nonequivalent structures. Moreover, we have checked that all the values obtained for the properties examined in the random structures of the L supercell are always inside the range obtained for the S supercell. An exhaustive comparison is not possible due to computational limitations.

The electronic energy (*E*) per formula unit of the calculated nonequivalent 16 structures of the 2 × 2 × 1 supercell span a range of approximately 0.15 eV and are collected in Table 1. Based on the small energetic differences between the 16 configurations, we expect a meaningful participation of most of the configurations in the observable properties of this compound even at temperatures below 300 K, as we will quantitatively discuss later in detail. To link the atomic distribution of the Cu^★^ atoms in the supercells with the calculated energy, an identification number (IDN) from 1 to 16 is associated with the four-letter code (see Figure 3). For example, the lowest energy configuration (1) is ADDA, whereas the one with the highest energy (16) is the ABCD structure.

**Table 1:** Summary of Calculated Properties per Formula Unit of the 16 Configurations of the 2 × 2 × 1 Supercell of Cu_4_TiTe_4_ ^a^

code	IDN (multiplicity)	SPG	*V*	Δ*E*	*E*_g_
ADDA	1 (8)	*P*2_1_	216.08	0.0000	0.1430
ADBC	2 (8)	*P*2_1_2_1_2	214.96	0.0016	0.5280
ADCB	3 (4)	*P*4̅2_1_ *m*	215.25	0.0050	0.3250
ACAC	4 (4)	*Pma*2	214.46	0.0134	0.2649
ABAB	5 (8)	*C*2	214.88	0.0165	0.3670
ACDA	6 (32)	*P*1	215.34	0.0183	0.3070
AAAA	7 (4)	*P*1	216.33	0.0197	0.0000
AABA	8 (32)	*P*1	215.59	0.0256	0.0819
ACBC	9 (32)	*P*1	214.63	0.0269	0.3680
ABDA	10 (32)	*P*1	215.06	0.0353	0.2669
AACA	11 (16)	*Cm*	215.28	0.0398	0.0609
ABCB	12 (16)	*Cm*	214.62	0.0479	0.3079
ACCA	13 (16)	*P*2	215.17	0.0619	0.1639
ABDC	14 (8)	*P*222_1_	214.07	0.0761	0.2649
ACBA	15 (32)	*P*1	214.46	0.0875	0.2050
ABCD	16 (4)	*P*4̅2*m*	213.38	0.1497	0.0209

a Volumes are given in Å^3^, and energies relative to the lowest energy configuration and band gaps in eV. The four-letters code, the multiplicities, and space groups (SPG) are also included. IDN refers to the identication number defined in the text.

To explore the different structural, energetic, and electronic properties these 16 configurations show, we plotted in Figures 4 and 5, respectively, their corresponding energy-volume curves per formula unit and the evolution of the static equilibrium volumes and band gaps of the configurations ordered by increasing energy. All the calculated equilibrium volumes are within a narrow interval, 214.86 ± 1.46 Å^3^, with values slightly above (3–6 Å^3^) the experimental volume at 296 ± 2 K,^1^ in concordance with typical errors of the PBE functional that tends to underbind the solids. The equilibrium volumes in Table 1 and Figure 4 are slightly different because the former ones come from the VASP geometry optimizations, while the figure shows the equilibrium values from the Vinet EOS fits.^31^ Cu_4_TiTe_4_ is a compressible material as evidenced by the low zero pressure bulk modulus (*B*_0_) of the 16 configurations, spanning a range between 36 and 41 GPa. Interestingly, the lowest energy configuration shows the second greatest volume whereas the higher energy configuration is the one with the lowest volume. As we will see later, this result has implications for the volume-related thermodynamic properties of this material.

**Figure 4:**
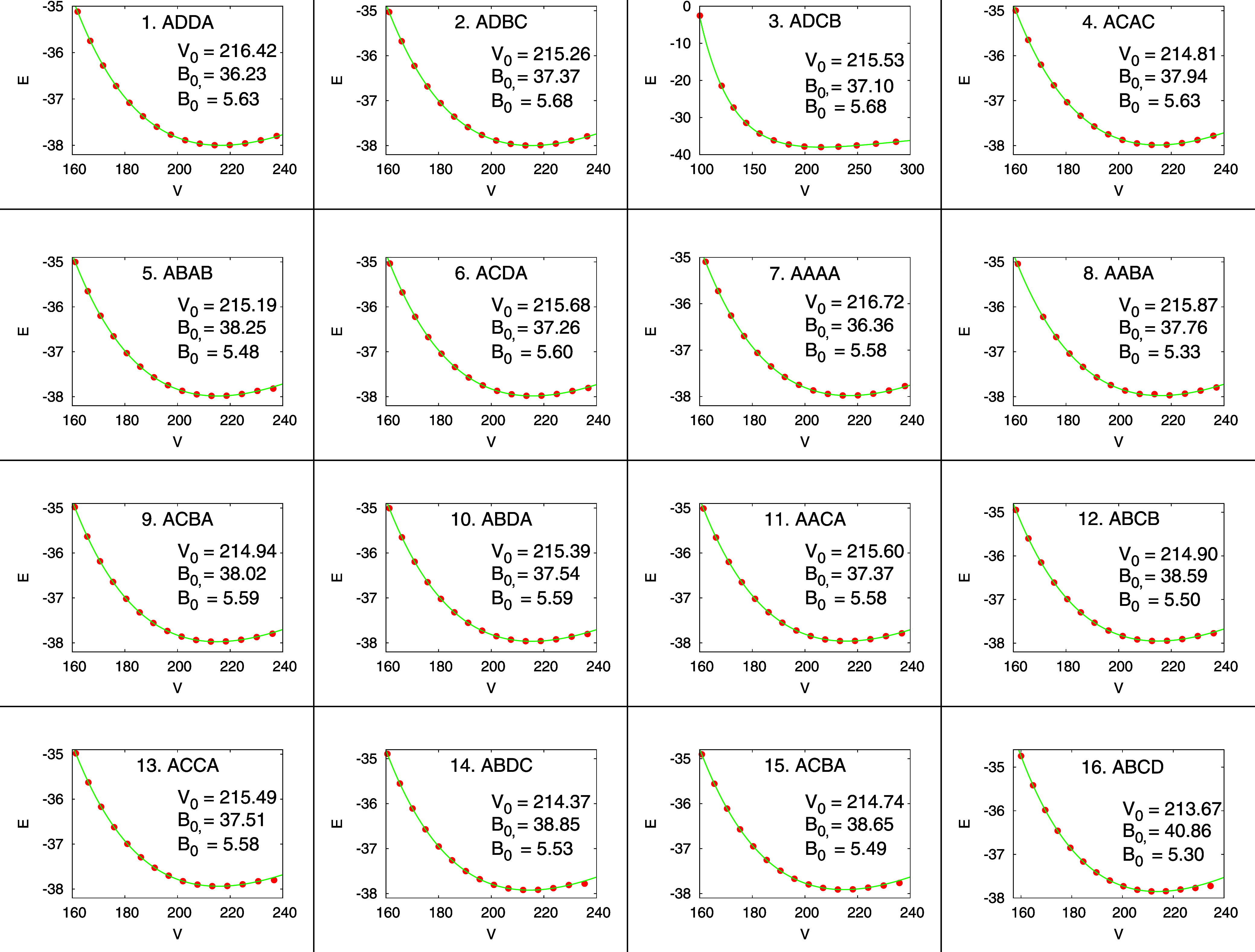
Equations of state of the 16 configurations of Cu_4_TiTe_4_ within the 2 × 2 × 1 supercell. EOS parameters (*V*_0_, *B*_0_, and *B*_0_^′^) are obtained by fittings to the analytical Vinet function.^31^ Energy is given in eV, Volume in Å^3^ and *B*_0_ in GPa.

**Figure 5:**
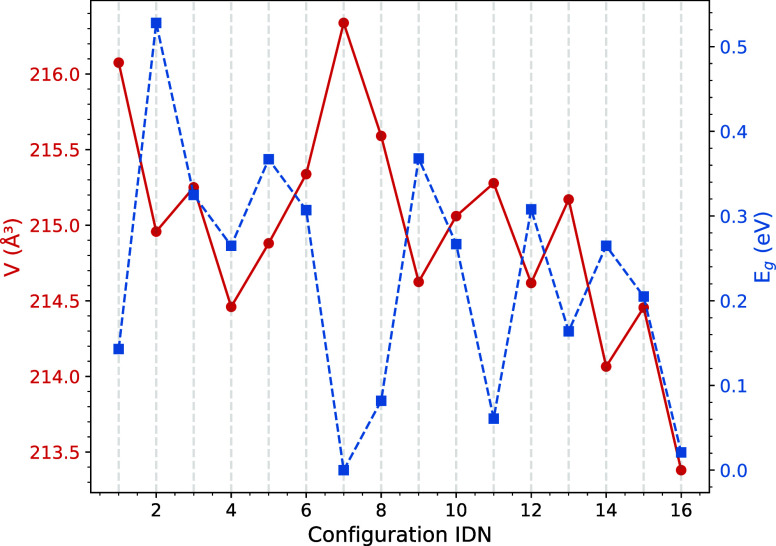
Calculated equilibrium volume and bandgap values of the 16 nonequivalent configurations of the 2 × 2 × 1 supercell of Cu_4_TiTe_4_. The configuration IDN refers to the identifiers in Table 1 and Figure 3.

We have selected four representative thermodynamic properties, volume (*V*_0_), thermal expansion coefficient (α), constant-pressure heat capacity (*C*_p_), and the zero pressure bulk modulus (*B*_0_) to check how the thermodynamic properties change as temperature increases in the 0–400 K range for each of the 16 nonequivalent configurations in Figure 6. In this Figure, we have emphasized the temperature evolution of these properties for three interesting configurations: the one with the lowest energy (ADDA), the one with a null band gap (AAAA), and the one called the average configuration (ABCD) that is the one with the highest energy as well as the one used as a model in the study of Lakshan et al.^1^ Our studied temperature range encompasses the one experimentally reported in ref 8, which extends from 200 to 400 K. We observe that *C*_p_ does not show meaningful differences between configurations, with all of them showing the same trend toward the classical Dulong and Petit limit (224.5 J·mol^–1^·K^–1^). A similar behavior is observed for α only in the low-temperature range. However, above 100 K, an increasing spread in α is observed and different values between ∼6 ×10^–5^ K^–1^ and 8.5×10^–5^ K^–1^ are predicted depending on the configuration. When *V*_0_(*T*) and *B*_0_(*T*) are analyzed in the 0–400 K interval, we find a similar range of values at a given temperature as the one discussed in the static approximation (see Table 1 and Figure 4), although the dependence of *B*_0_ on a particular configuration tends to increase at high temperature.

**Figure 6:**
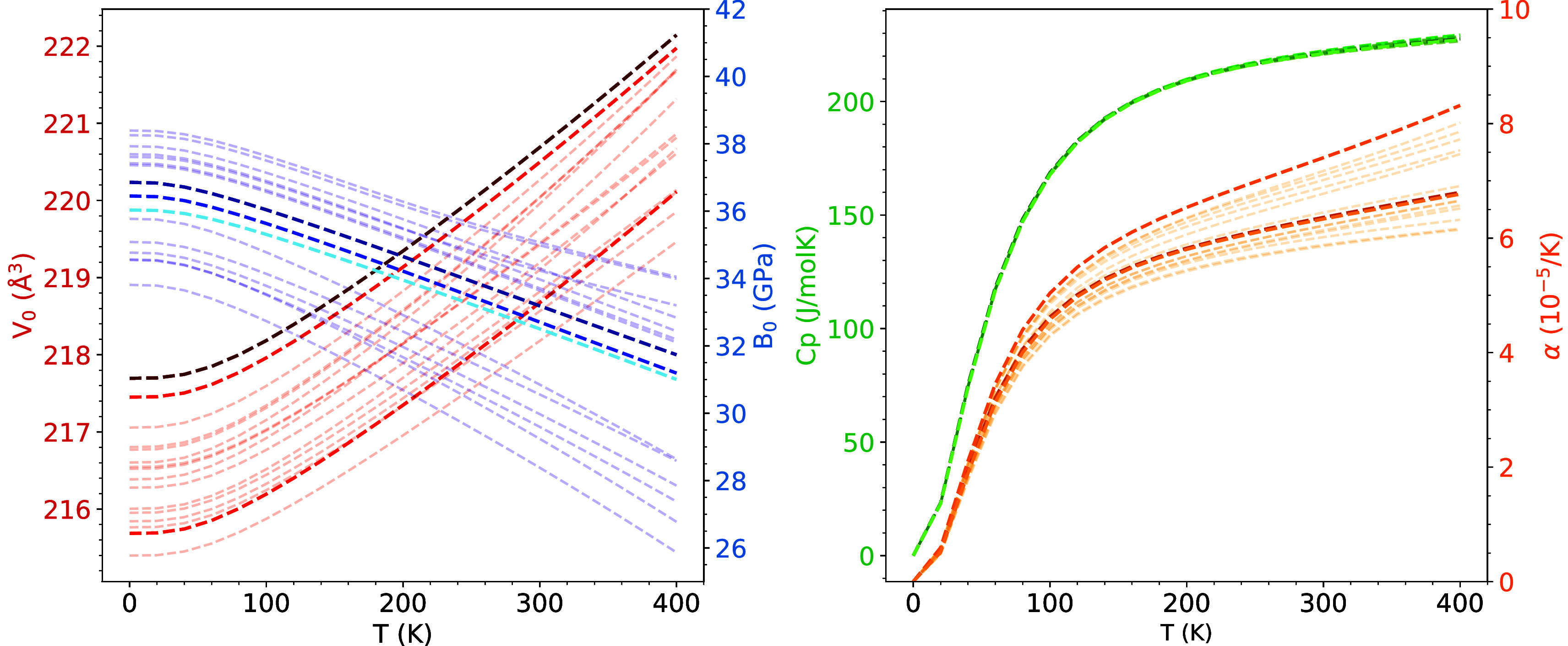
*V*_0_ and *B*_0_ (left panel), *C*_p_, and α (right panel) evolution with temperature of the 16 nonequivalent configurations of the 2 × 2 × 1 supercell. Results for configurations 1, 7, and 16 are colored with decreasing intensity.

Regardless of the variability of values exhibited by these 16 nonequivalent configurations, all the results shown in this subsection warn about the potential errors that may be made when a particular configuration is used as a reference to describe positional disordered crystals and the convenience of following a statistically weighted model to compute their average thermodynamic properties.

Given their potential technological application, the band gap (*E*_g_) and thermal conductivity (κ_L_) are two interesting properties worth calculating in this compound with positional disorder. Among the 16 configurations, the band gap is the property with the widest range of values oscillating between a semimetallic state in the AAAA configuration and around 0.5 eV in the ADBC structure. A saw-like pattern is seen in the evolution of both, band gap and volume as the configuration IDN increases from the lowest to the highest energy. Notice an almost opposite trend of both properties. Obviously, more data and systems should be explored to verify whether this general pattern deserves further analysis.

In the case of κ_L_, an accurate determination of its value would require a complex procedure involving the calculation of high-order anharmonic force constants as well as solving Boltzmann’s transport equation. Moreover, positional disorder introduces a non-negligible contribution to κ_L_ that must also be considered. For simplicity, we pursued an estimation of this property to evaluate the variability of the thermal conductivity along the set of the 16 nonequivalent configurations of our Cu_4_TiTe_4_ supercell. The use of approximate (yet reliable) equations, as those provided after exhaustive testing in refs 32,33 , allows us to explore if a particular configuration may be considered representative of this supercell model. In Figure 7, we show the estimated temperature evolution of the optical contribution and the total κ_L_ values using the Debye-Callaway model.^32^ As expected, the optical contribution to κ_L_ is almost negligible in this temperature range with values below 0.4 W·m^–1^·K^–1^ according to this approximated model. The range of κ_L_ values at 300 K is 10 ± 3 W·m^–1^·K^–1^ evidencing a narrow distribution of this property within the 16 configurations. A first conclusion from these results is that the structural (static) role played by Cu^★^ atoms is not crucial in the low κ_L_ values of this material. The large difference with the expected experimental value (κ_L_ = 0.19 W·m^–1^·K^–1^ in the related Cu_4_TiSe_4_ compound according to ref 3) is not only due to the approximations involved in the Debye-Callaway model used to estimate κ_L_, but also due to the existence of other (dynamic) mechanisms lowering the thermal conductivity. We again remark that the aim of our analyis at this regard is to check whether the range of κ_L_ values is large or narrow for the 16 nonequivalent configurations in order to provide insight on the role played by the atomic diffusion in this property.

**Figure 7:**
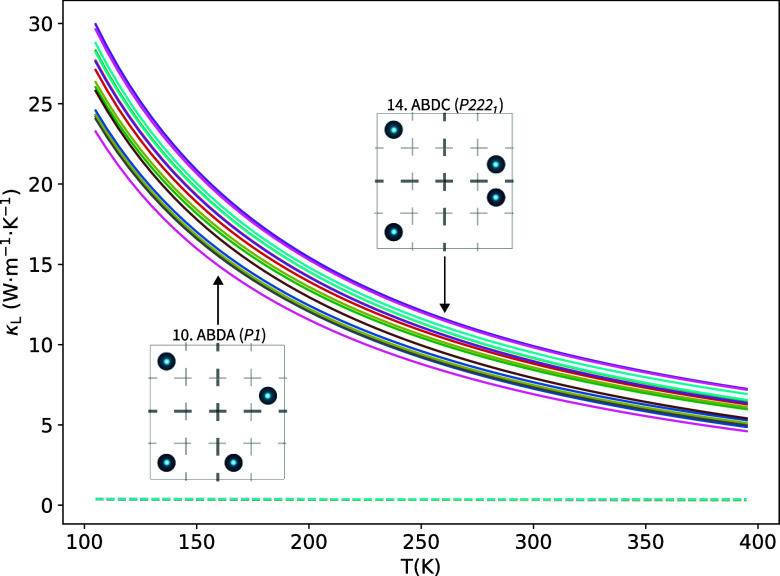
Estimated thermal conductivity using the Debye-Callaway model in the 100–400 K range for the 16 nonequivalent configurations of the 2 × 2 × 1 Cu_4_TiTe_4_ supercell. Total and optical contributions are represented as solid and dashed lines, respectively. Schematic configurations with the lowest and highest κ_L_ values are represented within the figure.

Notice that *E*_g_ and κ_L_ do not enter within the set of thermodynamic properties and, therefore, equations from section Thermodynamic Modeling of Positional Disorder do not apply to them. However, the exploration performed across the nonequivalent configurations provides information about the reliability of calculating these properties in a particular configuration. In the case of the *E*_g_ it is clear that the position of the Cu^★^ dramatically changes the electronic band structure, so static configurations play a role, and studying just one configuration may lead to erroneous results. However, this is not the case in κ_L_, where dynamic rather than static disorder controls the thermal flow across the sample. Interestingly enough, both cases highlight the importance of considering mesoscopic models to evaluate properties that are not susceptible to being averaged.

### Averaged Description of Static and Dynamic Positional Disorder in Cu_4_TiTe_4_ at Moderate Temperatures

We now follow the procedure outlined above to compute the thermodynamic properties of Cu_4_TiTe_4_ taking into account the positional disorder of Cu^★^. After computing DFT energies and QHA thermodynamic properties for the nonequivalent configurations of the 2 × 2 × 1 supercell, the next step requires the evaluation of the Boltzmann distribution of these 16 structures at different temperatures using eq 5. The DFT energies and degeneracies involved in this equation are collected in Table 1. In Figure 8, the population fractions (*w*_c_) between 100 and 900 K of the 16 configurations are associated with the sizes of the color bars ordered by their IDN number. Structures lower down in the bar have lower energies. Although there are some repetitions in the colors, this ordering avoids confusion. We have selected this temperature range since the synthesis of Cu_4_TiTe_4_ involves heating the reactants up to 900 K followed by quenching the sample to room conditions.^1^

**Figure 8:**
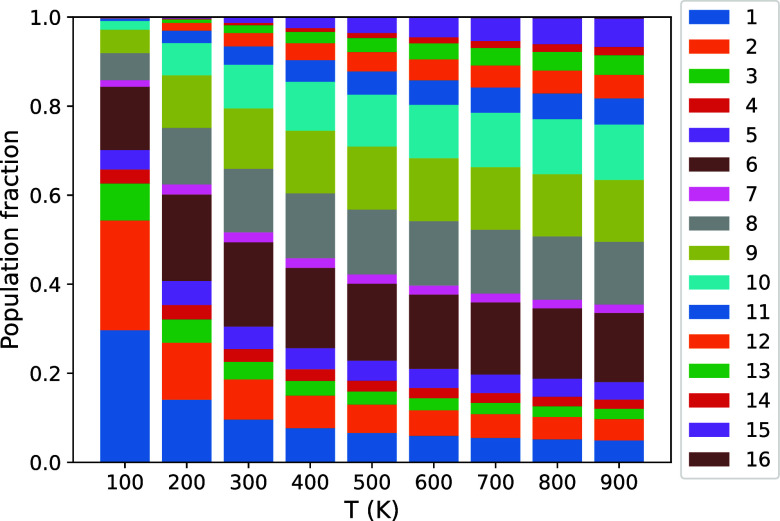
Population fraction at different temperatures for the 16 nonequivalent configurations obtained in the 2 × 2 × 1 supercell model of Cu_4_TiTe_4_. Structures lower down in the bar have lower energies.

This figure illustrates the importance of considering all the configurations involved in the 2 × 2 × 1 supercell. Due to the small energy difference between the nonequivalent configurations, we observe that all but the one with the highest energy have a significant statistical weight above 300 K and, even at 100 K, up to ten configurations should be taken into account when we evaluate the averaged thermodynamic properties. Specifically, we note that, from 200 K onward, the configuration with IDN = 6 (ACDA) has the highest contribution rather than the one with the lowest energy since ACDA has higher multiplicity (*g*_6_ = 32) and is only 0.0183 eV above the ground configuration (*g*_1_ = 8).

By applying eqs 7 and 8, and differentiating eq 2, we have computed the temperature evolution of *V*_0_, *B*_0_, α and *C*_p_ from 0 to 400 K, considering synthesis temperatures (*T*_s_) of 100, 200, 300, 400 and 900 K (solid lines in the top panels of Figure 9). We include the results obtained for the particular configurations with the lowest and highest energy (dashed lines). There are two important observations. First, the temperature evolution of these properties does not show significant changes with the synthesis temperatures. This is explained by resorting to the narrow energy distribution displayed by the nonequivalent configurations. This is a peculiarity of Cu_4_TiTe_4_ that should not be extrapolated to other disordered systems with wider energy distributions. Second, quantitative differences in the predictions of *V*_0_, *B*_0_, or α with respect to the results of particular configurations are apparent, although the static description trends remain very similar. The only property that does not show significant differences is *C*_p_, as expected from the analysis of the individual configurations.

**Figure 9:**
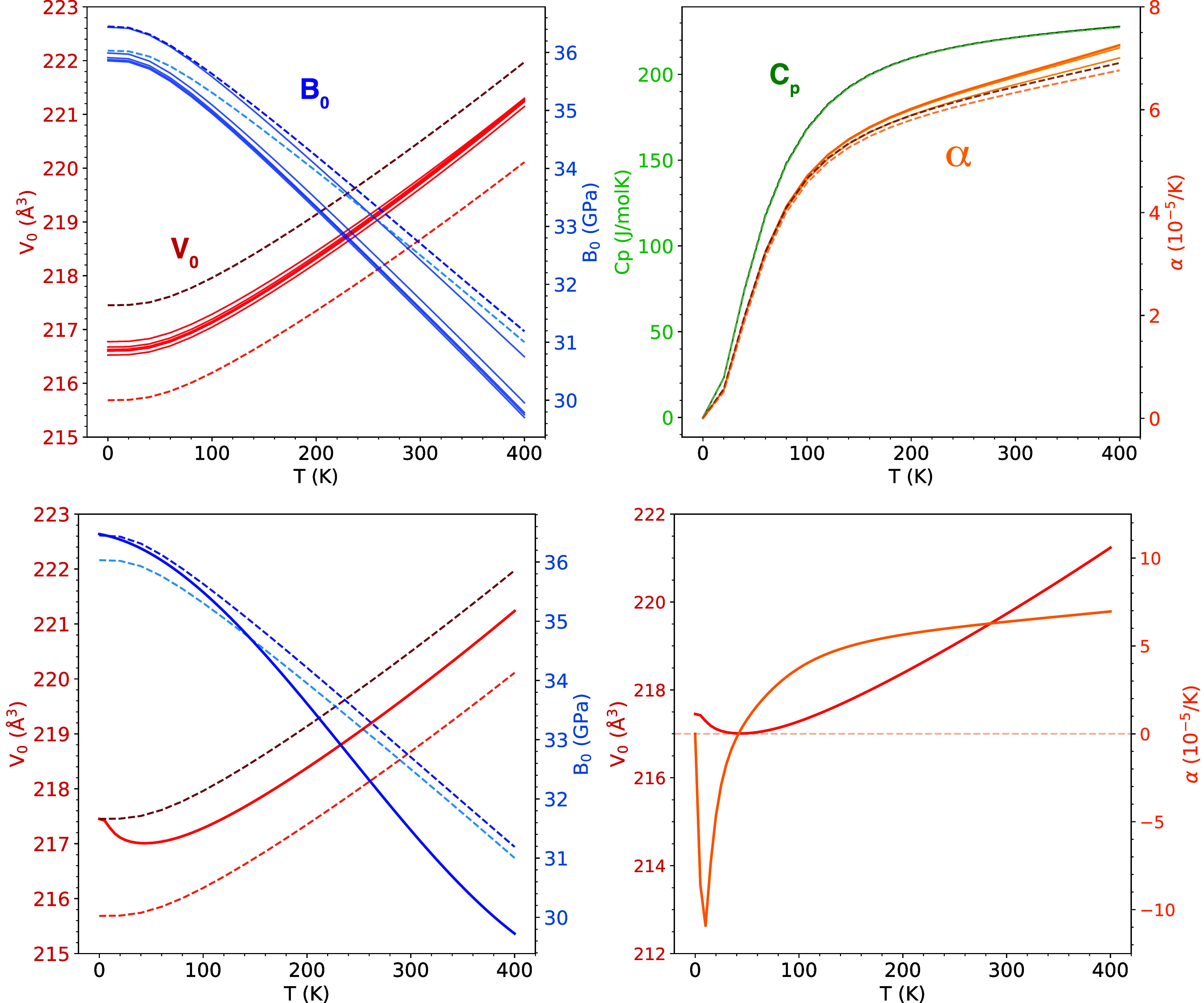
Top panel: temperature dependence of *V*_0_ and *B*_0_ (left figure) and α and *C*_*p*_ (right figure) according to the static positional disorder model (solid lines) at 100, 200, 300, 400, and 900 K and for the specific configurations with the lowest (lighter color) and highest energy (dashed lines). Bottom panel: Temperature dependence of *V*_0_ and *B*_0_ (left figure) and α and *V*_0_ (right figure) according to the dynamical positional disorder model (solid lines) and for the particular configurations with the lowest (lighter color) and highest energy (dashed lines).

In order to check if dynamic and static positional disorder coexist in the Cu_4_TiTe_4_ compound, we have calculated different Cu^★^ diffusion paths within the unit cell. The ADBC (IDN = 2) configuration has been taken as the reference structure to carry out this analysis using the NEB approach.^34^ We have chosen this one instead of the ground configuration since the IDN = 2 structure is the second one in the rank of energies and shows a higher symmetry than the lowest energy one. This selection eases the computational procedure. We expect similar results in the other configurations. We explore three different diffusion energy profiles of Cu^★^ from its starting A position in the ADBC configuration to B, C or D positions that lead to the BDBC (IDN = 15), CDBC (IDN = 12) or DDBC (IDN = 16) configurations, respectively.

The corresponding calculated diffusion energy profiles are shown in Figure 10. In all three atomic paths, the Cu^★^ movement can be split into two steps. The first one leads this atom to the central point of the cubic subcell (tetrahedral void of the Te atoms and also the center of the four equivalent 4*e* Wyckoff positions). In the second step, the Cu^★^ atom moves from this position to the final position. In other words, the diffusion path connects two equivalent 4e Wyckoff positions passing through the center of the tetrahedron they form. Notice that the atomic environments are not the same in the first and second steps resulting in asymmetric energy profiles. Results clearly inform about the feasibility of Cu^★^ jumping between the equivalent 4e Wyckoff positions since diffusion barriers as low as 0.4 eV are obtained (see Figure 10).

**Figure 10:**
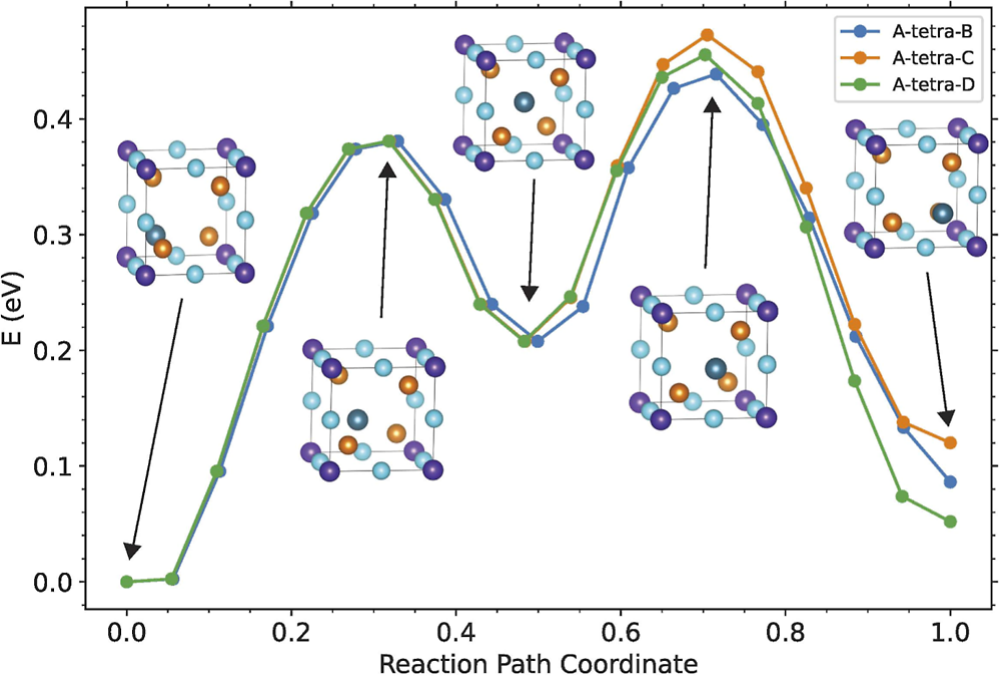
Energy profiles along the migration paths of the Cu^★^ atom from its nominal position A at the IDN = 2 configuration to positions B, C, and D corresponding to configurations with IDN = 15, 16, and 12, respectively. The copper atom moves in two steps. The center of the unit cell is the end of the first step and the beginning of the second step.

This result suggests the application of the more elaborate model obtained considering the dynamic positional disorder. Results are plotted in the bottom panel of Figure 9. Here we observe that the calculated temperature dependence of *B*_0_, *V*_0_, and α from this approach leads to quantitative and qualitative differences with respect to both particular configurations and the static disorder case (top panel of Figure 9). Two types of conclusions can be drawn: (i) in agreement with the static disorder analysis, we again observe that particular configurations are not necessarily representative of the system properties. Neither the most stable nor the more energetic configuration are able to reproduce the temperature dependence of these properties, and (ii) in contrast with the static disorder trend, only when the dynamic disorder is taken into account a negative thermal expansion (NTE) coefficient is predicted for Cu_4_TiTe_4_ at low temperatures. Although the reduction of the positional disorder as temperature decreases can yield a disorder–order phase transition^8^ likely preventing the observation of NTE behavior, we note that dynamic disorder may also uncover a qualitatively different thermodynamic picture. In summary, both the static and dynamic descriptions illustrate the necessity of including an accurate modeling of the positional disorder in real materials.

## Conclusions

A first-principles computational protocol has been proposed to evaluate thermodynamic properties in materials showing positional or so-called on-site atomic disorder. The strategy can be easily extended to deal with solid solutions and alloys. Both, static and dynamic disorders are addressed. The protocol is organized in three fundamental steps. The first step identifies the nonequivalent configurations and their degeneracies once the supercell size is defined. In the second step, standard electronic structure and QHA calculations are carried out in each nonequivalent configuration. Finally, averaged thermodynamic properties are obtained using Boltzmann fixed weights (static disorder) or temperature-dependent weights if dynamic disorder needs to be taken into account.

This strategy has been applied to Cu_4_TiTe_4_, an a priori unexpected disordered solid. In this compound, one Cu atom (denoted Cu^★^) is positionally disordered, occupying only one of the four Wyckoff positions assigned to it according to the *P*4̅3*m* space group determined in XRD experiments. 2 × 2 × 1 and 2 × 2 × 2 supercells containing up to 256 and 65,536 configurations, respectively, have been explored. The small supercell was used to test our computational strategy by evaluating a number of thermodynamic properties.

Unit cell volumes, heat capacities at constant pressure, thermal expansion coefficients, zero pressure bulk moduli, as well as band gaps, and thermal conductivities were computed for the 16 nonequivalent configurations of the 2 × 2 × 1 supercell. Properties that are not qualified to be averaged require mesoscopic models to provide accurate predictions. They show a meaningful variation in the case of the band gap (from metallic to band gaps of more than 0.5 eV) and span a lower range in the case of the thermal conductivity. Quantitative errors are observed if we choose to predict properties using a particular configuration. Moreover, the spread in the calculated values broadens with increasing temperature. Differences between the limiting values of the calculated ranges and the averaged (weighted) ones depend on the specific calculated property. *C*_p_ and α show a narrow range of values, whereas the dependence on the particular configuration is greater for *V*_0_ and *B*_0_. Due to the low energy barrier (less than 0.5 eV) associated with the diffusion of Cu^★^ among the four equivalent Wyckoff positions, we have considered the effect of dynamic disorder in the evaluation of the thermodynamic properties of Cu_4_TiTe_4_. Properties involving temperature derivatives are specially sensitive to dynamic disorder, as the thermal expansion coefficient evidence with a negative value a low temperature that was not captured by the static disorder scheme. We hope that our theoretical results motivate further experimental work to validate the interesting properties that we have evaluated for Cu_4_TiTe_4_.

## Supplementary Material



## Notes

Additional computational details of the static electronic structure calculations. Optimized structural parameters, atomic coordinates, and electronic energies for the 16 nonequivalent configurations. Additional computational details of the phonon calculations. Phonon density of states of the 16 nonequivalent configurations (PDF)
