# Supplementary material for: Solving Static and Dynamic Disorder in Cu4TiTe4: Crystal Structure and Thermodynamic Properties
Source: Inorg Chem. 2025 Feb 7;64(7):3223–34. doi: 10.1021/acs.inorgchem.4c04585 (PMC12571381; doi:10.1021/acs.inorgchem.4c04585)
Supplement: Supplementary file 1 [file ic4c04585_si_001.pdf]

Solving static and dynamic disorder in Cu_4TiTe_4: crystal structure and thermodynamic properties

Jorge Suárez-Recio,[†] Álvaro Lobato,[*,‡] Fernando Izquierdo-Ruiz,[‡] Ruth Franco,[¶] Alberto Otero-de-la-Roza,[¶] and J. Manuel Recio[*,¶]

†Instituto de Fusión Nuclear "Guillermo Velarde", Universidad Politécnica de Madrid, Madrid, E-28006, Spain and Departamento de Ingeniería Energética, Universidad Politécnica de Madrid, Madrid, E-28006, Spain

‡MALTA-Consolider Team and Departamento de Química Física, Universidad Complutense de Madrid, 28040 Madrid (Spain)

¶MALTA-Consolider Team and Departamento de Química Física y Analítica, Universidad de Oviedo, 33006 Oviedo (Spain)

* Corresponding authors: a.lobato@ucm.es; jmrecio@univoi.es

Table of Contents

Results **2**

Additional Electronic Structure Computational Details and Structural Data ...., ... 2

Additional Phonon Computational Details and Phonon Density of States 21

References **26**

Author Contributions **27**

Results

Additional Electronic Structure Computational Details and Structural Data

For full reproducibility of our results, we provide here a detailed account of the computational parameters and the resulting structural data for the sixteen non-equivalent Cu_4TiTe_4 configurations analyzed in the manuscript. As discussed therein, all calculations were performed using the Vienna Ab initio Simulation Package (VASP) [1], employing the projector-augmented wave (PAW) method [2], and the Perdew-Burke-Ernzerhof (PBE) exchange-correlation functional [3]. The kinetic energy cutoff for the plane-wave basis was set to 350 eV, ensuring accurate total energies and forces. A uniform Monkhorst-Pack **k**-point mesh [4] of 10×10×10 was employed for all configurations, providing well-converged Brillouin zone sampling and enabling consistent comparisons.

Structural optimizations were carried out until residual forces on every atom were below 1 meV/Å. The self-consistent field (SCF) cycles during these relaxations were considered converged when changes in the total energy were smaller than 1×10^{-8} eV. For each non-metallic configuration, we used ISMEAR = -5, which corresponds to the tetrahedron method with Blöch corrections for integrations in the reciprocal space, ensuring accurate total energies and avoiding artificial partial occupancies. For the single metallic configuration (IDN 7), a Methfessel-Paxton scheme (ISMEAR = 1) was employed to properly describe metallic states and capture the Fermi surface.

Table S1 presents the optimized lattice parameters *a*, *b*, and *c* (in Å), lattice angles α, β, and γ (in degrees), total energies per unit cell (in eV), and fractional coordinates (*x*, *y*, *z*) for all sixteen configurations. These data, combined with the computational details outlined above, ensure full reproducibility and provide a solid foundation for future theoretical and experimental investigations.

Table S01. Optimized structural parameters and atomic coordinates for the sixteen non-equivalent Cu_4TiTe_4 configurations discussed in the manuscript. The table includes the lattice constants *a*, *b*, and *c* (in Å), electronic energies (E) per formula unit (in eV), and the fractional atomic coordinates (*x*, *y*, *z*) for all atoms in the 2x2x1 supercell.

	**Lattice constants**				**Atomic Positions**			
**Configuration IDN**	*a* / *α*	*b* / *β*	*c* / *γ*	*E*	Atom type	*x*	*y*	*z*
**01**	12.07	11.87	6.03	-37.9969	Cu	0.13	0.13	0.27
	90.00	88.95	90.00		Cu	0.63	0.13	0.27
					Cu	0.36	0.63	0.72
					Cu	0.86	0.63	0.72

Cu	0.50	0.24	0.00
Cu	0.74	0.99	0.99
Cu	0.49	0.99	0.48
Cu	0.99	0.74	0.99
Cu	0.25	0.49	0.00
Cu	0.00	0.49	0.51
Cu	0.49	0.74	0.99
Cu	0.75	0.49	0.00
Cu	0.50	0.49	0.51
Cu	0.00	0.24	0.00
Cu	0.24	0.99	0.99
Cu	0.99	0.99	0.48
Ti	0.99	0.99	0.99
Ti	0.49	0.99	0.99
Ti	0.00	0.49	0.00
Ti	0.50	0.49	0.00
Te	0.36	0.12	0.25
Te	0.13	0.37	0.26
Te	0.37	0.36	0.75
Te	0.12	0.12	0.73
Te	0.86	0.12	0.25
Te	0.63	0.37	0.26
Te	0.87	0.36	0.75
Te	0.62	0.12	0.73
Te	0.37	0.62	0.26
Te	0.12	0.86	0.24
Te	0.36	0.87	0.73
Te	0.13	0.62	0.74
Te	0.87	0.62	0.26
Te	0.62	0.86	0.24
Te	0.86	0.87	0.73

					Te	0.63	0.62	0.74
**02**	11.99	11.91	6.02	-37.9953	Cu	0.14	0.14	0.28
	90.00	90.00	90.00		Cu	0.85	0.35	0.28
					Cu	0.35	0.64	0.71
					Cu	0.64	0.85	0.71
					Cu	0.50	0.25	0.01
					Cu	0.74	0.99	0.98
					Cu	0.49	0.99	0.51
					Cu	0.00	0.75	0.98
					Cu	0.25	0.50	0.98
					Cu	0.00	0.49	0.48
					Cu	0.50	0.75	0.99
					Cu	0.75	0.49	0.01
					Cu	0.50	0.50	0.51
					Cu	0.00	0.25	0.00
					Cu	0.24	0.00	0.01
					Cu	0.99	0.00	0.48
					Ti	0.99	0.00	0.99
					Ti	0.49	0.99	0.00
					Ti	0.00	0.49	0.99
					Ti	0.50	0.50	0.00
					Te	0.37	0.12	0.26
					Te	0.13	0.37	0.24
					Te	0.37	0.37	0.74
					Te	0.12	0.12	0.74
					Te	0.86	0.12	0.24
					Te	0.62	0.37	0.26
					Te	0.87	0.37	0.74
					Te	0.62	0.12	0.74
					Te	0.37	0.62	0.25
					Te	0.12	0.87	0.25

					Te	0.36	0.87	0.75
					Te	0.12	0.62	0.73
					Te	0.87	0.62	0.25
					Te	0.62	0.87	0.25
					Te	0.87	0.87	0.73
					Te	0.63	0.62	0.75
**03**	11.94	11.94	6.04	-37.992	Cu	0.14	0.14	0.26
	90.00	90.00	90.00		Cu	0.64	0.35	0.73
					Cu	0.35	0.64	0.73
					Cu	0.85	0.85	0.26
					Cu	0.50	0.25	0.00
					Cu	0.75	0.99	0.99
					Cu	0.50	0.00	0.50
					Cu	0.99	0.75	0.99
					Cu	0.25	0.50	0.00
					Cu	0.00	0.50	0.50
					Cu	0.49	0.74	0.00
					Cu	0.74	0.49	0.00
					Cu	0.50	0.50	0.52
					Cu	0.00	0.24	0.99
					Cu	0.24	0.00	0.99
					Cu	0.00	0.00	0.47
					Ti	0.00	0.00	0.98
					Ti	0.50	0.00	0.00
					Ti	0.00	0.50	0.00
					Ti	0.50	0.50	0.01
					Te	0.37	0.12	0.25
					Te	0.12	0.37	0.25
					Te	0.37	0.37	0.76
					Te	0.12	0.12	0.72
					Te	0.87	0.12	0.23

					Te	0.62	0.37	0.27
					Te	0.87	0.37	0.74
					Te	0.62	0.12	0.74
					Te	0.37	0.62	0.27
					Te	0.12	0.87	0.23
					Te	0.37	0.87	0.74
					Te	0.12	0.62	0.74
					Te	0.87	0.62	0.25
					Te	0.62	0.87	0.25
					Te	0.87	0.87	0.72
					Te	0.62	0.62	0.76
**04**	11.96	11.96	5.99	-37.9836	Cu	0.14	0.14	0.28
	90.00	90.00	90.00		Cu	0.85	0.35	0.28
					Cu	0.35	0.85	0.28
					Cu	0.64	0.64	0.28
					Cu	0.50	0.25	0.98
					Cu	0.75	0.00	0.98
					Cu	0.49	0.99	0.48
					Cu	0.00	0.75	0.98
					Cu	0.25	0.50	0.98
					Cu	0.99	0.49	0.48
					Cu	0.50	0.75	0.00
					Cu	0.75	0.50	0.00
					Cu	0.50	0.50	0.48
					Cu	0.00	0.25	0.00
					Cu	0.25	0.00	0.00
					Cu	0.00	0.00	0.48
					Ti	0.00	0.00	0.99
					Ti	0.49	0.99	0.99
					Ti	0.99	0.49	0.99
					Ti	0.50	0.50	0.99

					Te	0.37	0.13	0.25
					Te	0.13	0.37	0.25
					Te	0.37	0.37	0.73
					Te	0.13	0.13	0.74
					Te	0.86	0.12	0.25
					Te	0.62	0.36	0.25
					Te	0.86	0.36	0.74
					Te	0.62	0.12	0.73
					Te	0.36	0.62	0.25
					Te	0.12	0.86	0.25
					Te	0.36	0.86	0.74
					Te	0.12	0.62	0.73
					Te	0.87	0.63	0.25
					Te	0.63	0.87	0.25
					Te	0.87	0.87	0.73
					Te	0.63	0.63	0.74
**05**	11.91	11.99	6.02	-37.9804	Cu	0.13	0.14	0.28
	89.15	90.00	90.00		Cu	0.63	0.35	0.71
					Cu	0.13	0.85	0.71
					Cu	0.63	0.64	0.28
					Cu	0.49	0.25	0.98
					Cu	0.74	0.00	0.00
					Cu	0.49	0.00	0.50
					Cu	0.99	0.75	0.98
					Cu	0.24	0.50	0.00
					Cu	0.99	0.50	0.50
					Cu	0.49	0.74	0.01
					Cu	0.74	0.50	0.00
					Cu	0.50	0.50	0.50
					Cu	0.99	0.24	0.01
					Cu	0.24	0.00	0.00

					Cu	0.00	0.00	0.50
					Ti	0.99	0.00	0.00
					Ti	0.49	0.00	0.00
					Ti	0.99	0.50	0.00
					Ti	0.49	0.50	0.00
					Te	0.36	0.12	0.24
					Te	0.12	0.37	0.26
					Te	0.37	0.37	0.74
					Te	0.12	0.13	0.74
					Te	0.87	0.12	0.25
					Te	0.62	0.36	0.25
					Te	0.86	0.37	0.75
					Te	0.62	0.12	0.73
					Te	0.37	0.62	0.25
					Te	0.12	0.86	0.25
					Te	0.36	0.87	0.75
					Te	0.12	0.62	0.73
					Te	0.86	0.62	0.24
					Te	0.62	0.87	0.26
					Te	0.87	0.87	0.74
					Te	0.62	0.63	0.74
**06**	11.99	11.92	6.02	-37.9786	Cu	0.13	0.13	0.28
	90.00	89.50	89.60		Cu	0.63	0.14	0.26
					Cu	0.35	0.85	0.27
					Cu	0.85	0.63	0.72
					Cu	0.50	0.25	0.99
					Cu	0.74	0.00	0.98
					Cu	0.49	0.00	0.47
					Cu	0.99	0.74	0.99
					Cu	0.25	0.49	0.00
					Cu	0.99	0.49	0.51

					Cu	0.49	0.75	0.99
					Cu	0.75	0.50	0.00
					Cu	0.50	0.50	0.50
					Cu	0.00	0.24	0.01
					Cu	0.24	0.99	0.99
					Cu	0.00	0.99	0.49
					Ti	0.99	0.99	0.99
					Ti	0.49	0.00	0.98
					Ti	0.00	0.49	0.01
					Ti	0.50	0.50	0.00
					Te	0.36	0.13	0.24
					Te	0.13	0.37	0.26
					Te	0.37	0.37	0.74
					Te	0.12	0.12	0.74
					Te	0.86	0.12	0.25
					Te	0.63	0.37	0.25
					Te	0.87	0.36	0.75
					Te	0.62	0.12	0.72
					Te	0.37	0.62	0.26
					Te	0.12	0.86	0.24
					Te	0.37	0.87	0.73
					Te	0.13	0.62	0.75
					Te	0.87	0.62	0.26
					Te	0.62	0.87	0.23
					Te	0.87	0.87	0.73
					Te	0.62	0.62	0.74
**07**	12.01	12.01	6.00	-37.9772	Cu	0.13	0.13	0.27
	89.00	89.00	88.99		Cu	0.63	0.13	0.27
					Cu	0.13	0.63	0.27
					Cu	0.63	0.63	0.27
					Cu	0.49	0.24	0.99

Cu	0.74	0.99	0.99
Cu	0.49	0.99	0.49
Cu	0.99	0.74	0.99
Cu	0.24	0.49	0.99
Cu	0.99	0.49	0.49
Cu	0.49	0.74	0.99
Cu	0.74	0.49	0.99
Cu	0.49	0.49	0.49
Cu	0.99	0.24	0.99
Cu	0.24	0.99	0.99
Cu	0.99	0.99	0.49
Ti	0.99	0.99	0.99
Ti	0.49	0.99	0.99
Ti	0.99	0.49	0.99
Ti	0.49	0.49	0.99
Te	0.36	0.12	0.25
Te	0.12	0.36	0.25
Te	0.37	0.37	0.74
Te	0.12	0.12	0.73
Te	0.86	0.12	0.25
Te	0.62	0.36	0.25
Te	0.87	0.37	0.74
Te	0.62	0.12	0.73
Te	0.36	0.62	0.25
Te	0.12	0.86	0.25
Te	0.37	0.87	0.74
Te	0.12	0.62	0.73
Te	0.86	0.62	0.25
Te	0.62	0.86	0.25
Te	0.87	0.87	0.74
Te	0.62	0.62	0.73

**08**	11.95	11.99	6.01	-37.9714	Cu	0.13	0.13	0.27
	89.07	89.53	89.56		Cu	0.63	0.14	0.28
					Cu	0.13	0.63	0.27
					Cu	0.63	0.85	0.71
					Cu	0.49	0.25	0.01
					Cu	0.74	0.99	0.99
					Cu	0.50	0.00	0.50
					Cu	0.99	0.74	0.99
					Cu	0.24	0.50	0.00
					Cu	0.00	0.49	0.49
					Cu	0.49	0.75	0.99
					Cu	0.75	0.49	0.99
					Cu	0.49	0.50	0.50
					Cu	0.99	0.24	0.99
					Cu	0.24	0.00	0.00
					Cu	0.99	0.99	0.48
					Ti	0.99	0.99	0.99
					Ti	0.49	0.00	0.00
					Ti	0.00	0.49	0.99
					Ti	0.49	0.50	0.00
					Te	0.36	0.12	0.25
					Te	0.12	0.36	0.25
					Te	0.37	0.37	0.75
					Te	0.12	0.12	0.73
					Te	0.86	0.12	0.24
					Te	0.62	0.37	0.26
					Te	0.87	0.37	0.74
					Te	0.62	0.13	0.74
					Te	0.37	0.62	0.25
					Te	0.12	0.86	0.25
					Te	0.36	0.87	0.74

					Te	0.12	0.62	0.73
					Te	0.87	0.62	0.24
					Te	0.62	0.86	0.25
					Te	0.86	0.86	0.74
					Te	0.62	0.62	0.73
**09**	11.97	11.92	6.01	-37.97	Cu	0.14	0.14	0.28
	89.99	90.41	89.67		Cu	0.85	0.36	0.28
					Cu	0.35	0.85	0.28
					Cu	0.64	0.86	0.71
					Cu	0.49	0.25	0.99
					Cu	0.74	0.99	0.98
					Cu	0.49	0.99	0.50
					Cu	0.00	0.74	0.98
					Cu	0.25	0.50	0.98
					Cu	0.99	0.49	0.48
					Cu	0.50	0.75	0.00
					Cu	0.75	0.50	0.00
					Cu	0.50	0.50	0.50
					Cu	0.99	0.25	0.00
					Cu	0.24	0.00	0.01
					Cu	0.00	0.00	0.48
					Ti	0.99	0.00	0.99
					Ti	0.49	0.00	0.00
					Ti	0.00	0.49	0.99
					Ti	0.50	0.50	0.99
					Te	0.37	0.13	0.25
					Te	0.13	0.37	0.25
					Te	0.37	0.37	0.73
					Te	0.12	0.13	0.74
					Te	0.86	0.12	0.24
					Te	0.62	0.37	0.26

					Te	0.87	0.36	0.74
					Te	0.62	0.12	0.74
					Te	0.37	0.62	0.25
					Te	0.12	0.86	0.25
					Te	0.36	0.87	0.74
					Te	0.12	0.62	0.73
					Te	0.87	0.62	0.24
					Te	0.63	0.87	0.25
					Te	0.87	0.87	0.73
					Te	0.63	0.63	0.74
**10**	11.99	11.91	6.01	-37.9616	Cu	0.13	0.14	0.28
	89.58	89.52	89.97		Cu	0.63	0.13	0.27
					Cu	0.14	0.85	0.71
					Cu	0.85	0.63	0.71
					Cu	0.50	0.24	0.00
					Cu	0.74	0.00	0.00
					Cu	0.49	0.00	0.48
					Cu	0.99	0.74	0.98
					Cu	0.25	0.49	0.01
					Cu	0.99	0.50	0.51
					Cu	0.49	0.74	0.99
					Cu	0.75	0.49	0.99
					Cu	0.50	0.49	0.50
					Cu	0.00	0.24	0.01
					Cu	0.24	0.99	0.99
					Cu	0.99	0.99	0.49
					Ti	0.99	0.99	0.99
					Ti	0.49	0.00	0.99
					Ti	0.00	0.50	0.00
					Ti	0.50	0.49	0.00
					Te	0.36	0.12	0.24

					Te	0.12	0.37	0.26
					Te	0.37	0.37	0.75
					Te	0.12	0.13	0.74
					Te	0.86	0.12	0.25
					Te	0.63	0.37	0.25
					Te	0.87	0.36	0.75
					Te	0.62	0.12	0.73
					Te	0.37	0.62	0.26
					Te	0.12	0.86	0.25
					Te	0.37	0.87	0.73
					Te	0.13	0.62	0.74
					Te	0.87	0.62	0.25
					Te	0.62	0.87	0.25
					Te	0.86	0.87	0.74
					Te	0.62	0.62	0.73
**11**	11.97	11.97	6.00	-37.9572	Cu	0.13	0.13	0.26
	89.53	89.53	89.18		Cu	0.63	0.14	0.28
					Cu	0.14	0.63	0.28
					Cu	0.85	0.85	0.27
					Cu	0.50	0.24	0.00
					Cu	0.74	0.99	0.99
					Cu	0.50	0.00	0.49
					Cu	0.99	0.74	0.99
					Cu	0.24	0.50	0.00
					Cu	0.00	0.50	0.49
					Cu	0.49	0.74	0.99
					Cu	0.74	0.49	0.99
					Cu	0.49	0.49	0.50
					Cu	0.00	0.24	0.98
					Cu	0.24	0.00	0.98
					Cu	0.99	0.99	0.47

					Ti	0.99	0.99	0.98
					Ti	0.49	0.00	0.99
					Ti	0.00	0.49	0.99
					Ti	0.49	0.49	0.00
					Te	0.36	0.12	0.25
					Te	0.12	0.36	0.25
					Te	0.37	0.37	0.75
					Te	0.12	0.12	0.72
					Te	0.86	0.13	0.24
					Te	0.62	0.37	0.26
					Te	0.87	0.37	0.74
					Te	0.62	0.12	0.74
					Te	0.37	0.62	0.26
					Te	0.13	0.86	0.24
					Te	0.37	0.87	0.74
					Te	0.12	0.62	0.74
					Te	0.86	0.62	0.25
					Te	0.62	0.86	0.25
					Te	0.87	0.87	0.73
					Te	0.62	0.62	0.74
**12**	11.94	11.94	6.02	-37.949	Cu	0.14	0.14	0.27
	89.58	90.42	89.99		Cu	0.63	0.36	0.72
					Cu	0.14	0.85	0.71
					Cu	0.85	0.85	0.27
					Cu	0.50	0.25	0.99
					Cu	0.74	0.99	0.00
					Cu	0.49	0.00	0.50
					Cu	0.99	0.75	0.99
					Cu	0.25	0.50	0.01
					Cu	0.99	0.50	0.50
					Cu	0.49	0.74	0.01

					Cu	0.74	0.49	0.99
					Cu	0.50	0.49	0.51
					Cu	0.00	0.25	0.00
					Cu	0.24	0.00	0.99
					Cu	0.00	0.99	0.48
					Ti	0.00	0.99	0.99
					Ti	0.49	0.00	0.00
					Ti	0.99	0.50	0.00
					Ti	0.49	0.50	0.00
					Te	0.37	0.13	0.25
					Te	0.12	0.37	0.26
					Te	0.36	0.37	0.75
					Te	0.12	0.13	0.73
					Te	0.87	0.12	0.24
					Te	0.62	0.37	0.26
					Te	0.87	0.37	0.74
					Te	0.62	0.12	0.74
					Te	0.37	0.62	0.26
					Te	0.13	0.86	0.25
					Te	0.37	0.87	0.74
					Te	0.12	0.62	0.74
					Te	0.86	0.62	0.25
					Te	0.62	0.87	0.26
					Te	0.86	0.87	0.73
					Te	0.62	0.63	0.75
**13**	12.03	11.88	6.01	-37.935	Cu	0.13	0.14	0.27
	90.00	90.00	89.18		Cu	0.63	0.14	0.27
					Cu	0.36	0.85	0.27
					Cu	0.86	0.85	0.27
					Cu	0.50	0.25	0.99
					Cu	0.75	0.00	0.98

Cu	0.50	0.00	0.47
Cu	0.99	0.74	0.99
Cu	0.25	0.50	0.00
Cu	0.00	0.50	0.50
Cu	0.49	0.74	0.99
Cu	0.75	0.50	0.00
Cu	0.50	0.50	0.50
Cu	0.00	0.25	0.99
Cu	0.25	0.00	0.98
Cu	0.00	0.00	0.47
Ti	0.00	0.00	0.98
Ti	0.50	0.00	0.98
Ti	0.00	0.50	0.00
Ti	0.50	0.50	0.00
Te	0.36	0.13	0.24
Te	0.13	0.37	0.26
Te	0.37	0.37	0.75
Te	0.12	0.12	0.73
Te	0.86	0.13	0.24
Te	0.63	0.37	0.26
Te	0.87	0.37	0.75
Te	0.62	0.12	0.73
Te	0.36	0.62	0.26
Te	0.13	0.86	0.24
Te	0.37	0.87	0.73
Te	0.12	0.62	0.75
Te	0.86	0.62	0.26
Te	0.63	0.86	0.24
Te	0.87	0.87	0.73
Te	0.62	0.62	0.75

**14**	11.92	11.95	6.00	-37.9208	Cu	0.14	0.14	0.28
	90.00	90.00	90.00		Cu	0.85	0.35	0.28
					Cu	0.14	0.85	0.71
					Cu	0.85	0.64	0.71
					Cu	0.50	0.25	0.99
					Cu	0.74	0.00	0.00
					Cu	0.49	0.00	0.50
					Cu	0.00	0.75	0.98
					Cu	0.25	0.50	0.00
					Cu	0.99	0.50	0.50
					Cu	0.50	0.75	0.00
					Cu	0.75	0.50	0.00
					Cu	0.50	0.50	0.50
					Cu	0.00	0.25	0.01
					Cu	0.24	0.00	0.00
					Cu	0.00	0.00	0.50
					Ti	0.99	0.00	0.00
					Ti	0.49	0.00	0.00
					Ti	0.00	0.50	0.00
					Ti	0.50	0.50	0.00
					Te	0.37	0.13	0.25
					Te	0.13	0.37	0.25
					Te	0.37	0.37	0.74
					Te	0.12	0.13	0.74
					Te	0.86	0.12	0.25
					Te	0.62	0.36	0.25
					Te	0.87	0.36	0.74
					Te	0.62	0.12	0.74
					Te	0.37	0.62	0.25
					Te	0.12	0.86	0.25
					Te	0.37	0.86	0.74

					Te	0.13	0.62	0.74
					Te	0.87	0.63	0.25
					Te	0.62	0.87	0.25
					Te	0.86	0.87	0.74
					Te	0.62	0.63	0.74
**15**	11.98	11.90	6.01	-37.9095	Cu	0.13	0.14	0.27
	89.59	89.57	89.63		Cu	0.63	0.14	0.27
					Cu	0.35	0.85	0.27
					Cu	0.64	0.85	0.71
					Cu	0.50	0.25	0.00
					Cu	0.74	0.00	0.98
					Cu	0.49	0.99	0.48
					Cu	0.99	0.74	0.99
					Cu	0.24	0.49	0.99
					Cu	0.00	0.49	0.50
					Cu	0.49	0.75	0.99
					Cu	0.75	0.50	0.00
					Cu	0.50	0.50	0.50
					Cu	0.00	0.24	0.00
					Cu	0.24	0.99	0.00
					Cu	0.99	0.00	0.48
					Ti	0.99	0.99	0.99
					Ti	0.49	0.99	0.99
					Ti	0.00	0.49	0.00
					Ti	0.50	0.50	0.00
					Te	0.36	0.13	0.25
					Te	0.13	0.37	0.26
					Te	0.37	0.37	0.74
					Te	0.12	0.12	0.73
					Te	0.86	0.12	0.24
					Te	0.63	0.37	0.26

					Te	0.87	0.37	0.74
					Te	0.62	0.13	0.73
					Te	0.36	0.62	0.25
					Te	0.12	0.86	0.25
					Te	0.36	0.87	0.73
					Te	0.12	0.62	0.74
					Te	0.87	0.62	0.25
					Te	0.63	0.86	0.25
					Te	0.87	0.87	0.73
					Te	0.63	0.62	0.74
**16**	11.92	11.92	6.00	-37.8473	Cu	0.14	0.14	0.27
	90.00	90.00	90.00		Cu	0.85	0.14	0.72
					Cu	0.14	0.85	0.72
					Cu	0.85	0.85	0.27
					Cu	0.50	0.25	0.00
					Cu	0.75	0.00	0.00
					Cu	0.50	0.00	0.50
					Cu	0.00	0.75	0.00
					Cu	0.25	0.50	0.00
					Cu	0.00	0.50	0.50
					Cu	0.50	0.74	0.00
					Cu	0.74	0.50	0.00
					Cu	0.50	0.50	0.50
					Cu	0.00	0.24	0.00
					Cu	0.24	0.00	0.00
					Cu	0.00	0.00	0.50
					Ti	0.00	0.00	0.00
					Ti	0.50	0.00	0.00
					Ti	0.00	0.50	0.00
					Ti	0.50	0.50	0.00
					Te	0.37	0.13	0.25

	Te	0.13	0.37	0.25
	Te	0.37	0.37	0.74
	Te	0.13	0.13	0.74
	Te	0.86	0.13	0.25
	Te	0.62	0.37	0.25
	Te	0.86	0.37	0.74
	Te	0.62	0.13	0.74
	Te	0.37	0.62	0.25
	Te	0.13	0.86	0.25
	Te	0.37	0.86	0.74
	Te	0.13	0.62	0.74
	Te	0.86	0.62	0.25
	Te	0.62	0.86	0.25
	Te	0.86	0.86	0.74
	Te	0.62	0.62	0.74

Additional Phonon Computational Details and Phonon Density of States

To further verify the dynamical stability of the optimized Cu_4TiTe_4 configurations discussed above, we evaluated their phonon density of states (DOS) using *phonopy* [5], as described in the main text. The phonon calculations were performed on 2×2×2 supercells generated from the conventional unit cell of each configuration. The SCF convergence, integration method of the Brillouin zone, and the k-point mesh used in the phonon calculations were the same as those described above. Because the chosen conventional cell depends on the configuration's symmetry, the total number of atoms in the supercell can vary. As shown in Figure S01, the absence of imaginary frequencies confirms the dynamical stability of these configurations.

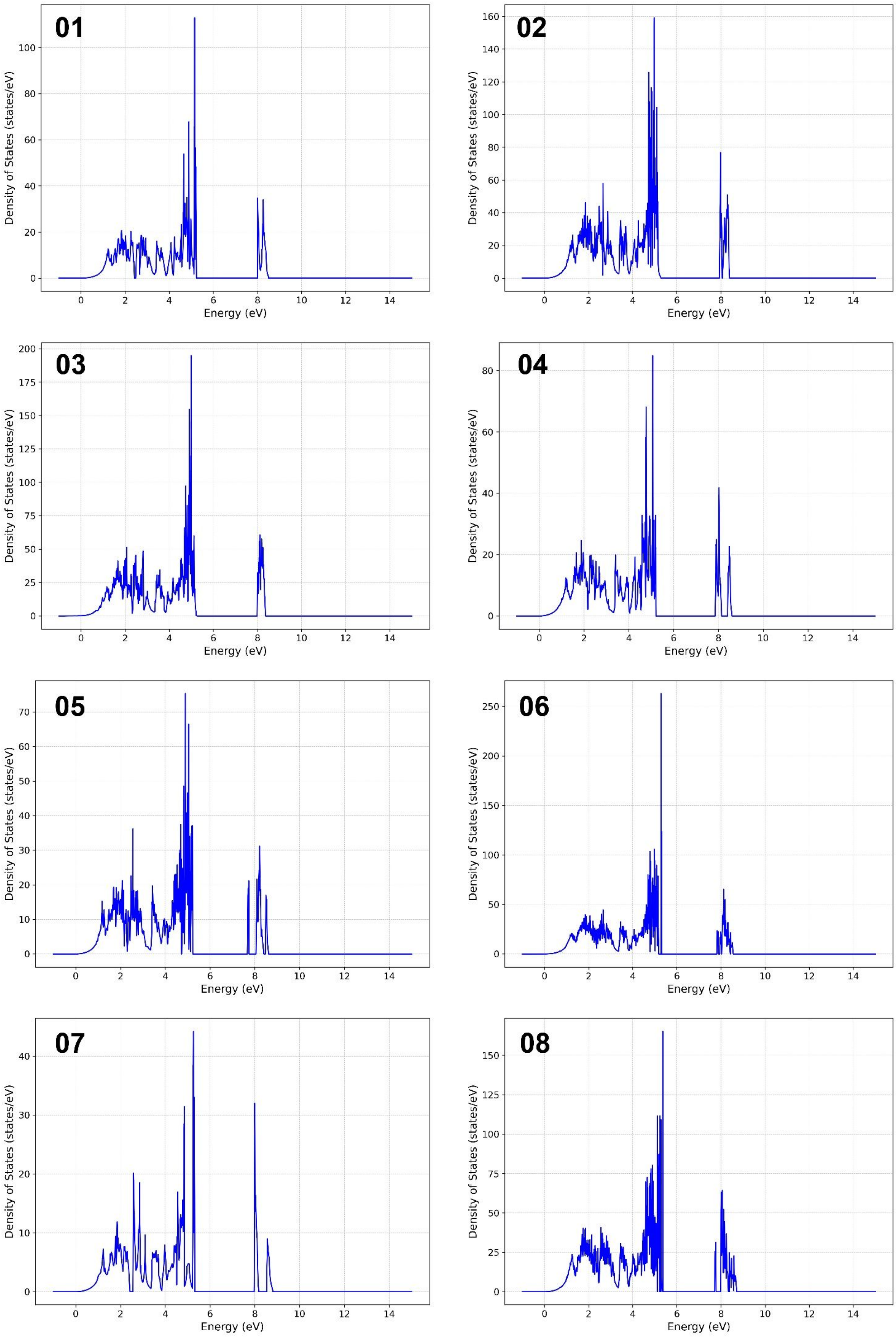

Figure S01. Phonon DOS for configurations IDN = 01-08.

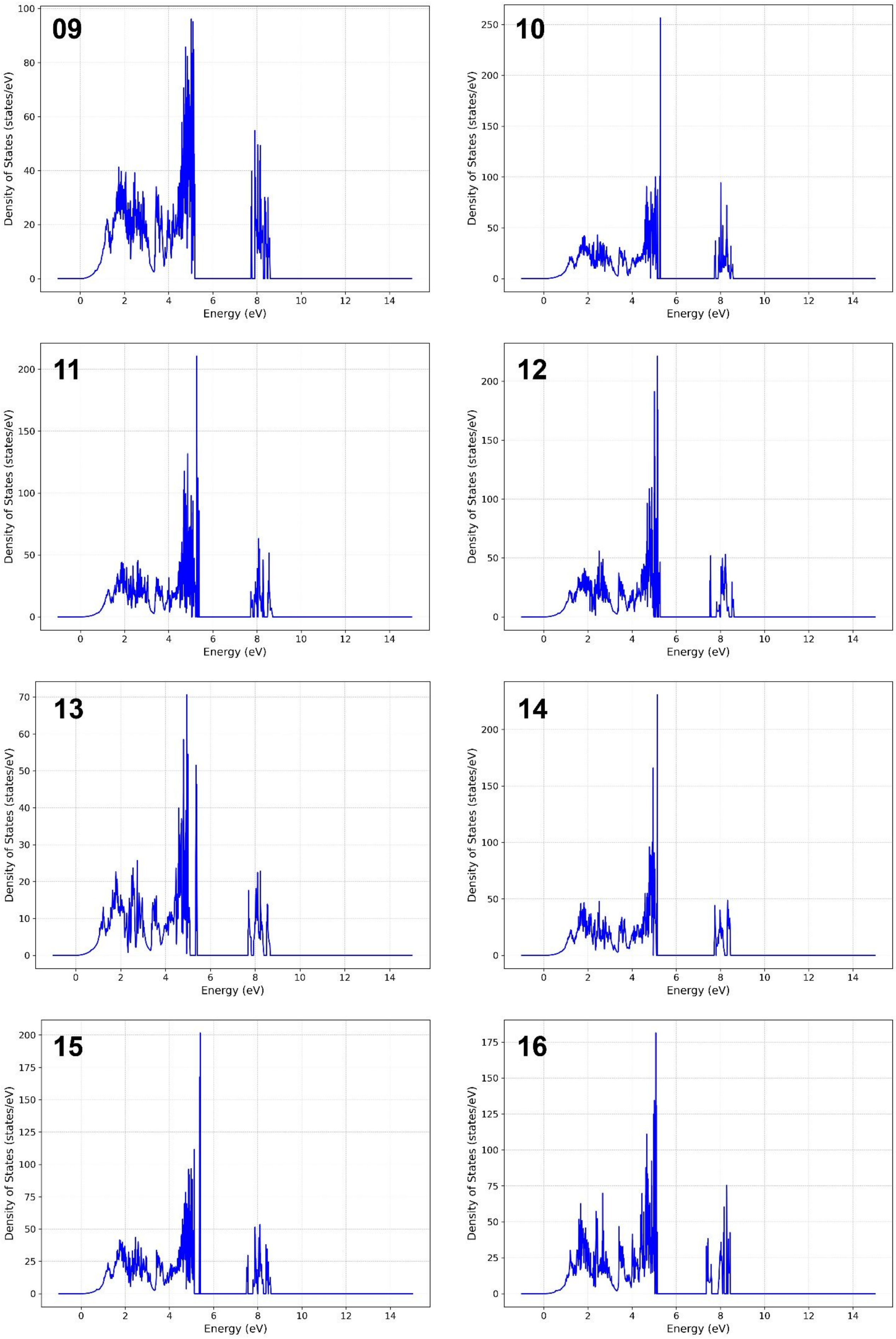

Figure S01 (continued). Phonon DOS for configurations IDN = 09-16.

References

[1] Kresse, G.; Hafner, J. Ab initio molecular dynamics for liquid metals. Phys. Rev. B 1993, **47**, 558–561.
[2] Blöchl, P. E. Projector augmented-wave method. Phys. Rev. B 1994, **50**, 17953–17979.
[3] Perdew, J. P.; Burke, K.; Ernzerhof, M. Generalized Gradient Approximation Made Simple. Phys. Rev. Lett. 1996, **77**, 3865–3868.
[4] Monkhorst, H.J.; Pack, J.D. Special points for Brillouin-zone integrations. Phys. Rev. B 1976, **13**, 5188-5192
[5] Togo, A.; Chaput, L.; Tadano, T.; Tanaka, I. Implementation strategies in phonopy and phono3py. J. Phys.: Condens. Matter 2023, **35**, 353001

Author Contributions

J.M.R., A.L., and F.I.R designed research; J.S.R., A.L, R.F, and A.O.R. performed the calculations; J.M.R., A.L., F.I., and A.O.R. wrote the original draft of the paper; J.M.R., A.L., R.F., F.I.R., J.S.R., and A.O.R. revised and edited the paper.
